# Supplementary material for: Effects of Contagious Respiratory Pathogens on Breath Biomarkers
Source: Antioxidants (Basel). 2024 Jan 29;13(2):172. doi: 10.3390/antiox13020172 (PMC10886173; doi:10.3390/antiox13020172)
Supplement: Supplementary file 1 [file antioxidants-13-00172-s001.zip › antioxidants-2790219-supplementary.pdf]

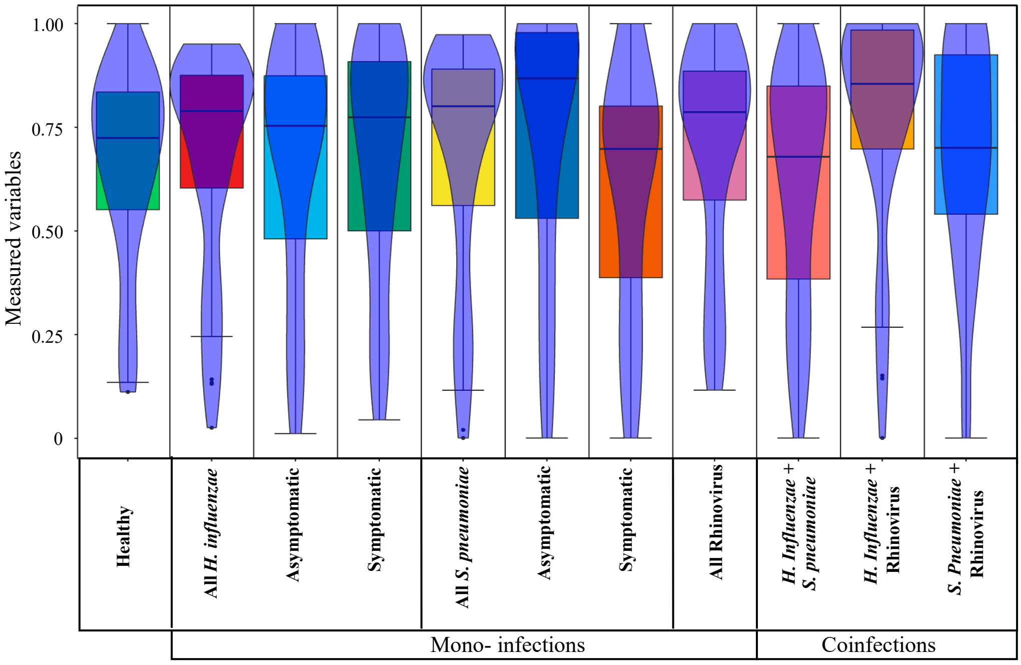

Figure S1: Violin plots based on measured variables presented in the heat map. Group and sub-group wise cumulative distribution of normalised variables from Figure 1 is presented here. Plots are depicting overall expressions of measured independent variables (VOCs) in each group and between multiple groups.

Table S1: Sum formulas and protonated masses of potentially exogenous VOCs

Sum formula	Mass
(C3H4)H+	41.034
(C3H6)H+	43.046
(CH2O2)H+	47.017
(C3H4O)H+	57.041
(C4H8)H+	57.072
(C5H6)H+	67.057
(C5H10)H+	71.082
(C6H6)H+	79.051
(C6H8)H+	81.070
(C6H10)H+	83.079
(C5H8O)H+	85.059
(C6H12)H+	85.103
(C7H8)H+	93.054
(C6H6O)H+	95.055
(C6H8O)H+	97.089
(C8H8)H+	105.063
(C8H10)H+	107.064
(C8H12)H+	109.092
(C7H10O)H+	111.100
(C9H10)H+	119.077
(C8H8O)H+	121.062
(C7H6O2)H+	123.044
(C9H10O)H+	135.070
(C10H12O)H+	149.056
(C10H22O)H+	159.094

Table S2: Overview for all 44 quantified VOCs with group-wise median and percentiles in the expiration.

EXPIRATIONS

Healthy; Mono-pathogens

Sum formula	Mass	Healthy (n=256)			All *H. Influenzae* (n=97)			Asymptomatic *H. Influenzae* (n=55)			Symptomatic *H. Influenzae* (n=42)		
		Median	25-percentile	75-percentile	Median	25-percentile	75-percentile	Median	25-percentile	75-percentile	Median	25-percentile	75-percentile
(CH2O)H+	31.018	113.60	81.88	167.55	117.89	91.54	174.91	122.51	87.68	167.47	117.01	96.10	176.26
CH4O)H+	33.031	95.26	58.61	146.64	90.25	57.49	128.47	95.71	70.71	130.29	78.38	43.85	127.31
(H2S)H+	34.970	2.47	2.16	2.79	2.54	2.31	2.97	2.49	2.29	2.83	2.60	2.36	3.16
(C3H4)H+	41.034	12256.76	7528.37	21146.61	12446.73	7309.96	20943.27	12501.16	7420.82	20674.29	12186.12	6290.89	20920.59
(C3H6)H+	43.046	13792.42	7916.82	23649.62	13954.17	7403.38	25262.56	13286.54	7483.44	25217.31	14311.66	7088.21	25645.96
(CO2)H+	44.997	1557.05	1348.71	1760.00	1626.42	1362.66	1814.33	1661.97	1419.58	1859.09	1547.02	1285.19	1796.01
(C2H4O)H+	45.027	59.10	42.94	87.57	56.05	40.39	87.78	55.35	40.88	87.39	57.41	40.15	88.79
(CH2O2)H+	47.017	82.87	68.29	105.53	83.01	70.48	99.58	83.41	70.97	97.14	82.48	70.46	106.02
(C2H6O)H+	47.056	< LOQ	< LOQ	48.41	< LOQ	< LOQ	< LOQ	< LOQ	< LOQ	< LOQ	< LOQ	< LOQ	< LOQ
(CH4S)H+	49.011	13.87	10.02	20.30	13.24	10.11	19.70	12.53	9.91	18.46	13.78	10.67	20.78
(C3H4O)H+	57.041	< LOQ	< LOQ	0.24	< LOQ	< LOQ	0.22	< LOQ	< LOQ	0.27	< LOQ	< LOQ	0.09
(C4H8)H+	57.072	100.51	70.79	133.40	99.58	72.00	122.20	104.71	82.09	130.57	87.24	69.89	114.74
(C3H6O)H+	59.049	474.02	351.58	648.27	543.76	406.72	733.79	570.37	421.19	745.08	509.85	370.94	717.28
(C2H4O2)H+	61.031	12.39	9.38	16.99	13.27	10.67	18.89	12.22	10.55	18.01	13.62	11.38	19.10
(C3H8O)H+	61.066	1780.30	1127.75	2792.16	1931.83	1250.74	3239.32	1794.73	1193.81	3325.48	2063.83	1304.39	2985.25
(C2H6S)H+	63.025	23.61	10.45	58.16	20.99	8.74	55.12	27.86	12.49	59.92	14.30	< LOQ	46.98
(C5H6)H+	67.057	52.91	37.74	71.71	53.37	39.75	75.09	57.14	43.95	81.37	48.76	36.00	65.70
(C5H8)H+	69.070	101.96	72.47	139.18	108.16	69.15	144.64	116.14	79.22	173.21	92.68	63.77	135.96
(C4H6O)H+	71.043	0.54	0.41	0.87	0.58	0.45	0.90	0.58	0.45	0.93	0.58	0.43	0.86
(C5H10)H+	71.082	19.76	15.03	26.01	20.49	15.34	24.85	22.05	17.07	26.10	16.80	14.16	23.73
(C4H8O)H+	73.067	< LOQ	< LOQ	1.99	< LOQ	< LOQ	11.51	< LOQ	< LOQ	20.25	< LOQ	< LOQ	< LOQ
(C6H6)H+	79.051	0.86	0.38	2.67	0.95	0.37	2.20	0.96	0.31	3.24	0.94	0.38	1.77
(C6H8)H+	81.070	62.35	40.36	103.93	68.01	43.67	104.54	72.73	49.09	109.12	62.06	34.57	87.81
(C6H10)H+	83.079	< LOQ	< LOQ	10.09	< LOQ	< LOQ	9.76	7.49	< LOQ	10.23	< LOQ	< LOQ	8.22
(C5H8O)H+	85.059	7.72	6.49	9.24	7.96	6.81	10.56	8.00	7.06	10.86	7.90	6.24	9.75
(C6H12)H+	85.103	8.77	6.33	11.07	9.25	6.47	11.19	9.62	7.01	11.35	8.12	6.32	10.94
(C5H10O)H+	87.047	55.62	36.18	86.39	56.46	33.55	76.04	65.19	43.09	82.10	46.32	31.63	61.53
(C4H8S)H+	89.046	1.26	0.85	2.20	1.23	0.87	2.30	1.26	0.89	2.54	1.07	0.84	2.13
(C4H10S)H+	91.056	1.47	1.18	1.99	1.54	1.14	1.91	1.61	1.14	1.94	1.45	1.12	1.91
(C7H8)H+	93.054	< LOQ	< LOQ	2.92	< LOQ	< LOQ	3.19	< LOQ	< LOQ	4.87	< LOQ	< LOQ	< LOQ
(C6H6O)H+	95.055	6.18	4.40	8.36	5.67	4.39	7.98	5.75	4.32	8.42	5.40	4.54	7.47
(C6H8O)H+	97.089	17.64	11.11	28.16	18.76	10.95	24.63	19.48	11.67	29.22	17.64	9.18	20.48
(C6H12O)H+	101.059	< LOQ	< LOQ	< LOQ	< LOQ	< LOQ	< LOQ	< LOQ	< LOQ	< LOQ	< LOQ	< LOQ	< LOQ
(C8H8)H+	105.063	21.82	16.60	27.53	22.19	16.83	26.64	21.75	17.28	25.05	23.54	16.10	29.54
(C8H10)H+	107.064	< LOQ	< LOQ	< LOQ	< LOQ	< LOQ	< LOQ	< LOQ	< LOQ	< LOQ	< LOQ	< LOQ	< LOQ
(C8H12)H+	109.092	1.98	1.49	2.64	1.91	1.35	2.80	2.05	1.54	2.92	1.66	0.30	2.33
(C7H10O)H+	111.100	15.93	< LOQ	22.14	16.19	< LOQ	22.60	16.69	< LOQ	22.22	8.10	< LOQ	23.98
(C9H10)	119.077	< LOQ	< LOQ	26.56	< LOQ	< LOQ	29.63	< LOQ	< LOQ	26.02	22.42	< LOQ	30.21
(C8H8O)H+	121.062	26.82	< LOQ	37.95	24.91	< LOQ	35.34	24.32	< LOQ	34.67	26.35	< LOQ	34.95
(C7H6O2)H+	123.044	14.43	11.15	19.06	14.22	11.46	18.76	15.39	12.19	22.58	12.85	11.16	17.01
(C9H10O)H+	135.070	17.41	13.83	21.97	18.12	14.10	22.37	15.83	13.71	21.00	18.93	14.80	22.92
(C10H16)H+	137.133	20.07	12.20	33.68	22.62	13.98	37.99	23.86	16.22	38.88	20.43	12.44	36.28
(C10H12O)H+	149.056	< LOQ	< LOQ	14.00	< LOQ	< LOQ	14.92	< LOQ	< LOQ	17.03	< LOQ	< LOQ	13.38
(C10H22O)H+	159.094	< LOQ	< LOQ	14.47	< LOQ	< LOQ	18.32	< LOQ	< LOQ	16.50	12.56	< LOQ	20.14

Mono-pathogens (continued)

Sum formula	Mass	All *S. pneumoniae* (n=40)			Asymptomatic *S. pneumoniae* (n=25)			Symptomatic *S. pneumoniae* (n=15)			All Rhinovirus (n=34)		
		Median	25-percentile	75-percentile	Median	25-percentile	75-percentile	Median	25-percentile	75-percentile	Median	25-percentile	75-percentile
(CH2O)H+	31.018	130.29	95.65	195.80	129.61	96.05	192.01	132.80	99.93	221.61	155.89	73.37	219.81
CH4O)H+	33.031	86.91	56.88	126.08	80.97	55.20	124.39	93.88	58.92	118.77	98.24	55.52	126.60
(H2S)H+	34.970	2.51	2.35	2.77	2.43	2.24	2.54	2.76	2.51	2.92	2.73	2.45	2.94
(C3H4)H+	41.034	13798.87	8289.09	25724.20	18483.05	8872.23	28104.29	12635.69	6551.25	17590.89	12958.36	4189.24	27796.22
(C3H6)H+	43.046	15659.53	8944.29	32656.23	21988.11	9780.13	35322.11	13643.92	7391.93	20000.28	14533.23	4679.09	34734.30
(CO2)H+	44.997	1578.91	1324.23	1719.88	1640.02	1485.64	1937.61	1327.73	1287.90	1560.91	1520.38	1418.35	1754.79
(C2H4O)H+	45.027	62.33	44.88	101.17	84.96	45.15	104.83	57.43	43.36	64.83	56.69	42.61	104.58
(CH2O2)H+	47.017	80.06	68.93	96.79	83.25	71.83	96.64	69.55	66.34	96.63	84.54	65.50	114.68
(C2H6O)H+	47.056	< LOQ	< LOQ	< LOQ	< LOQ	< LOQ	< LOQ	< LOQ	< LOQ	< LOQ	< LOQ	< LOQ	46.51
(CH4S)H+	49.011	15.81	10.55	22.18	17.83	12.47	22.18	11.39	9.14	19.28	15.14	11.56	19.55
(C3H4O)H+	57.041	< LOQ	< LOQ	0.89	< LOQ	< LOQ	1.12	< LOQ	< LOQ	0.07	< LOQ	< LOQ	0.35
(C4H8)H+	57.072	107.87	68.06	129.82	117.20	75.98	129.46	103.21	66.74	128.08	102.20	76.69	118.56
(C3H6O)H+	59.049	570.47	434.34	801.87	590.28	435.60	805.86	483.54	436.88	728.56	483.42	334.23	805.39
(C2H4O2)H+	61.031	14.67	11.02	19.31	13.72	10.68	19.08	16.91	11.75	23.50	13.33	11.38	22.27
(C3H8O)H+	61.066	2400.23	1329.47	3831.68	2769.63	1260.63	4568.00	2232.24	1428.74	2857.43	2067.88	728.31	4331.07
(C2H6S)H+	63.025	38.11	9.60	69.24	46.82	13.15	86.90	13.06	< LOQ	46.90	18.90	6.35	59.76
(C5H6)H+	67.057	51.69	41.81	65.70	49.83	42.75	63.12	52.30	40.35	69.84	51.47	40.25	62.37
(C5H8)H+	69.070	91.31	71.18	151.35	104.84	81.28	163.99	80.92	66.52	123.16	96.22	72.66	113.73
(C4H6O)H+	71.043	0.52	0.41	0.70	0.56	0.41	1.03	0.52	0.42	0.64	0.64	0.45	1.14
(C5H10)H+	71.082	21.12	15.82	28.02	22.34	15.36	30.69	19.61	16.19	22.96	21.88	17.78	25.50
(C4H8O)H+	73.067	< LOQ	< LOQ	4.66	< LOQ	< LOQ	< LOQ	< LOQ	< LOQ	11.20	< LOQ	< LOQ	10.26
(C6H6)H+	79.051	0.87	0.39	1.56	0.88	0.40	1.34	0.68	0.41	2.36	1.10	0.57	1.93
(C6H8)H+	81.070	76.99	53.03	138.43	75.06	53.46	142.60	81.09	39.59	115.39	70.77	45.96	159.04
(C6H10)H+	83.079	< LOQ	< LOQ	10.73	< LOQ	< LOQ	19.48	< LOQ	< LOQ	8.79	< LOQ	< LOQ	8.40
(C5H8O)H+	85.059	7.85	6.59	9.74	8.32	6.62	10.28	7.78	6.76	9.19	7.77	7.01	8.98
(C6H12)H+	85.103	9.74	6.69	11.36	9.50	7.20	12.16	10.03	6.46	10.39	8.66	7.72	11.04
(C5H10O)H+	87.047	71.07	51.01	115.87	80.16	56.00	121.29	56.34	40.19	79.79	51.48	40.01	76.22
(C4H8S)H+	89.046	1.21	0.94	2.11	1.71	0.94	2.71	1.13	0.98	1.23	1.60	0.96	3.24
(C4H10S)H+	91.056	1.38	1.20	1.98	1.37	1.11	2.13	1.39	1.32	1.65	1.53	1.21	2.04
(C7H8)H+	93.054	< LOQ	< LOQ	3.72	< LOQ	< LOQ	3.66	< LOQ	< LOQ	3.77	< LOQ	< LOQ	3.41
(C6H6O)H+	95.055	7.32	5.38	9.14	7.38	5.56	9.08	6.36	5.21	9.05	6.38	4.69	10.98
(C6H8O)H+	97.089	18.64	12.54	28.48	20.71	13.98	33.69	17.51	10.07	24.62	14.24	11.18	21.93
(C6H12O)H+	101.059	< LOQ	< LOQ	< LOQ	< LOQ	< LOQ	< LOQ	< LOQ	< LOQ	< LOQ	< LOQ	< LOQ	< LOQ
(C8H8)H+	105.063	21.23	17.58	29.37	25.83	18.79	31.61	18.36	15.81	22.42	22.58	18.54	26.95
(C8H10)H+	107.064	< LOQ	< LOQ	< LOQ	< LOQ	< LOQ	< LOQ	< LOQ	< LOQ	< LOQ	< LOQ	< LOQ	< LOQ
(C8H12)H+	109.092	1.85	1.54	2.89	1.90	1.60	2.90	1.65	1.36	2.38	2.19	1.55	2.76
(C7H10O)H+	111.100	18.68	< LOQ	26.22	19.46	< LOQ	26.62	< LOQ	< LOQ	21.82	19.89	< LOQ	24.70
(C9H10)	119.077	< LOQ	< LOQ	27.34	23.64	< LOQ	29.58	< LOQ	< LOQ	12.17	< LOQ	< LOQ	26.52
(C8H8O)H+	121.062	29.26	< LOQ	42.74	30.47	< LOQ	44.45	28.96	< LOQ	34.38	29.67	< LOQ	34.97
(C7H6O2)H+	123.044	15.02	12.66	20.02	16.12	13.08	19.57	14.27	11.95	18.92	15.27	11.72	20.13
(C9H10O)H+	135.070	20.43	14.63	21.50	19.91	14.81	22.26	20.56	11.93	21.07	20.11	15.49	23.73
(C10H16)H+	137.133	27.56	16.76	44.17	27.47	16.81	44.63	27.66	16.99	37.88	26.77	16.25	76.89
(C10H12O)H+	149.056	5.87	< LOQ	15.30	11.73	< LOQ	15.01	< LOQ	< LOQ	16.46	< LOQ	< LOQ	15.56
(C10H22O)H+	159.094	< LOQ	< LOQ	20.46	< LOQ	< LOQ	20.21	< LOQ	< LOQ	20.47	13.21	< LOQ	22.97

Co-pathogens

Sum formula	Mass	*H. influenzae* + *S. pneumoniae* (n=24)			*H. influenzae* + Rhinovirus (n=16)			*S. pneumoniae* + Rhinovirus (n=12)		
		Median	25-percentile	75-percentile	Median	25-percentile	75-percentile	Median	25-percentile	75-percentile
(CH2O)H+	31.018	142.02	112.20	182.95	143.74	95.08	196.71	125.26	89.77	165.09
CH4O)H+	33.031	74.55	56.67	160.94	76.66	53.39	169.71	91.46	64.10	129.98
(H2S)H+	34.970	2.68	2.38	2.88	2.49	2.28	2.77	2.61	2.26	2.93
(C3H4)H+	41.034	15671.86	11173.19	22452.41	16238.48	7469.02	21386.17	14517.52	9471.70	21324.80
(C3H6)H+	43.046	16175.51	11748.45	27073.66	18348.13	8392.25	26234.25	17176.58	8798.22	25351.76
(CO2)H+	44.997	1529.24	1359.86	1647.63	1601.89	1526.31	1826.98	1611.85	1427.70	1715.26
(C2H4O)H+	45.027	65.08	45.44	91.18	73.18	50.08	103.93	65.52	52.62	74.35
(CH2O2)H+	47.017	84.43	68.79	113.79	85.19	71.34	113.80	82.36	68.44	91.82
(C2H6O)H+	47.056	< LOQ	< LOQ	146.77	< LOQ	< LOQ	53.03	< LOQ	< LOQ	43.15
(CH4S)H+	49.011	15.73	10.09	19.99	12.52	11.14	21.73	14.43	10.26	16.98
(C3H4O)H+	57.041	< LOQ	< LOQ	0.21	< LOQ	< LOQ	0.95	< LOQ	< LOQ	0.08
(C4H8)H+	57.072	93.07	81.80	124.71	103.88	85.17	133.22	87.71	48.31	122.76
(C3H6O)H+	59.049	512.06	366.76	698.74	485.34	416.91	663.91	462.52	366.46	559.16
(C2H4O2)H+	61.031	14.02	10.56	18.13	13.98	11.05	18.55	12.01	9.37	13.69
(C3H8O)H+	61.066	1897.82	1467.03	3042.21	2492.68	1237.72	3791.26	2275.75	927.84	2671.99
(C2H6S)H+	63.025	20.22	3.41	59.36	50.60	14.49	63.22	25.33	11.32	49.77
(C5H6)H+	67.057	47.18	30.97	67.52	52.78	41.18	83.36	48.23	35.03	61.54
(C5H8)H+	69.070	82.60	49.30	138.76	100.15	77.70	190.05	98.47	59.11	112.81
(C4H6O)H+	71.043	0.50	0.38	0.64	0.68	0.53	1.23	0.41	0.33	0.46
(C5H10)H+	71.082	19.97	16.05	24.35	21.96	17.69	27.14	18.53	10.87	24.17
(C4H8O)H+	73.067	< LOQ	< LOQ	8.99	< LOQ	< LOQ	1.92	< LOQ	< LOQ	14.04
(C6H6)H+	79.051	0.52	0.27	1.50	1.31	0.70	3.07	0.85	0.43	1.89
(C6H8)H+	81.070	60.88	42.27	88.39	92.40	56.73	135.40	63.07	47.43	90.11
(C6H10)H+	83.079	< LOQ	< LOQ	8.43	< LOQ	< LOQ	10.87	< LOQ	< LOQ	< LOQ
(C5H8O)H+	85.059	7.58	5.90	9.68	8.43	7.17	10.41	7.50	5.49	8.99
(C6H12)H+	85.103	8.50	7.10	11.52	8.95	7.44	11.27	7.57	5.64	10.61
(C5H10O)H+	87.047	52.71	36.84	90.32	62.40	44.69	99.56	59.94	32.38	68.95
(C4H8S)H+	89.046	0.91	0.69	1.85	1.36	0.94	2.76	0.90	0.62	1.21
(C4H10S)H+	91.056	1.52	1.09	1.96	1.47	1.37	1.69	1.11	0.99	1.68
(C7H8)H+	93.054	< LOQ	< LOQ	< LOQ	< LOQ	< LOQ	3.59	< LOQ	< LOQ	1.08
(C6H6O)H+	95.055	5.12	4.27	7.42	6.02	5.20	7.89	6.15	5.16	9.97
(C6H8O)H+	97.089	14.03	9.49	21.64	21.35	17.19	26.22	17.29	9.41	21.24
(C6H12O)H+	101.059	< LOQ	< LOQ	< LOQ	< LOQ	< LOQ	< LOQ	< LOQ	< LOQ	< LOQ
(C8H8)H+	105.063	17.96	14.25	30.85	21.95	17.36	28.23	21.27	13.97	29.24
(C8H10)H+	107.064	< LOQ	< LOQ	< LOQ	< LOQ	< LOQ	< LOQ	< LOQ	< LOQ	< LOQ
(C8H12)H+	109.092	1.72	1.31	2.31	2.06	1.70	2.69	1.82	1.64	2.19
(C7H10O)H+	111.100	8.81	< LOQ	22.30	17.29	< LOQ	22.54	7.94	< LOQ	31.73
(C9H10)	119.077	< LOQ	< LOQ	25.22	< LOQ	< LOQ	23.48	11.51	< LOQ	27.98
(C8H8O)H+	121.062	27.63	< LOQ	32.39	27.04	19.14	38.73	12.36	< LOQ	28.91
(C7H6O2)H+	123.044	13.84	11.25	16.77	15.42	13.89	19.92	12.96	11.51	17.27
(C9H10O)H+	135.070	16.57	14.06	20.65	17.01	13.90	20.46	18.34	12.08	22.84
(C10H16)H+	137.133	23.79	14.18	37.39	32.88	22.52	50.00	26.21	19.48	32.02
(C10H12O)H+	149.056	< LOQ	< LOQ	13.46	< LOQ	< LOQ	16.93	< LOQ	< LOQ	13.06
(C10H22O)H+	159.094	13.92	< LOQ	22.50	6.40	< LOQ	16.16	5.91	< LOQ	13.49

Table S3: Overview for all 44 quantified VOCs with group-wise median and percentiles in the room air.

ROOM AIR					Mono-infections								
Sum formula	Mass	Healthy (n=256)			All *H. Influenzae* (n=97)			Asymptomatic *H. Influenzae* (n=55)			Symptomatic *H. Influenzae* (n=42)		
		Median	25-percentile	75-percentile	Median	25-percentile	75-percentile	Median	25-percentile	75-percentile	Median	25-percentile	75-percentile
(CH2O)H+	31.018	173.75	86.57	265.60	177.47	86.82	276.64	172.17	96.54	288.38	188.27	78.50	273.88
CH4O)H+	33.031	< LOQ	< LOQ	< LOQ	< LOQ	< LOQ	< LOQ	< LOQ	< LOQ	< LOQ	< LOQ	< LOQ	< LOQ
(H2S)H+	34.970	2.76	2.42	3.26	2.93	2.47	3.34	2.95	2.43	3.33	2.89	2.63	3.58
(C3H4)H+	41.034	16594.13	7032.04	27312.52	16435.90	6841.33	27291.92	16779.99	9001.47	26607.49	14932.08	6522.85	28211.95
(C3H6)H+	43.046	11602.23	5379.68	19293.46	11662.42	5379.68	20057.78	12161.91	6519.19	20057.78	11200.69	5180.41	20187.02
(CO2)H+	44.997	43.47	31.64	64.24	41.44	26.50	57.50	42.36	29.87	50.73	38.48	20.26	65.82
(C2H4O)H+	45.027	50.35	26.45	89.59	53.36	26.93	91.15	55.44	34.55	84.73	50.89	24.92	96.30
(CH2O2)H+	47.017	44.98	40.68	50.78	45.49	40.97	50.78	45.49	40.24	51.97	45.99	41.41	48.86
(C2H6O)H+	47.056	< LOQ	< LOQ	< LOQ	< LOQ	< LOQ	< LOQ	< LOQ	< LOQ	< LOQ	< LOQ	< LOQ	< LOQ
(CH4S)H+	49.011	6.79	5.53	7.77	6.48	5.59	7.97	6.44	5.56	7.70	6.54	5.64	8.72
(C3H4O)H+	57.041	< LOQ	< LOQ	< LOQ	< LOQ	< LOQ	< LOQ	< LOQ	< LOQ	< LOQ	< LOQ	< LOQ	< LOQ
(C4H8)H+	57.072	110.51	79.30	147.76	110.71	54.35	148.25	100.07	55.64	143.40	120.50	59.90	151.75
(C3H6O)H+	59.049	8.23	3.34	16.45	8.39	3.86	16.14	8.65	4.25	15.81	8.11	3.00	17.73
(C2H4O2)H+	61.031	13.74	9.85	17.53	14.91	9.75	17.49	14.01	8.87	17.16	15.73	11.26	19.20
(C3H8O)H+	61.066	636.51	358.34	1302.68	797.03	325.45	1341.20	839.04	329.83	1304.32	613.21	325.45	1390.59
(C2H6S)H+	63.025	< LOQ	< LOQ	18.95	< LOQ	< LOQ	16.50	< LOQ	< LOQ	15.32	< LOQ	< LOQ	21.77
(C5H6)H+	67.057	12.76	9.14	16.14	11.71	8.87	14.89	11.29	9.06	14.02	13.32	8.89	16.30
(C5H8)H+	69.070	0.21	< LOQ	2.85	< LOQ	< LOQ	2.19	< LOQ	< LOQ	0.75	0.83	< LOQ	2.55
(C4H6O)H+	71.043	0.35	0.26	0.48	0.35	0.25	0.46	0.32	0.24	0.45	0.38	0.26	0.47
(C5H10)H+	71.082	19.76	13.36	24.77	19.07	11.16	24.15	17.63	11.16	23.81	20.05	11.39	24.15
(C4H8O)H+	73.067	21.51	< LOQ	52.52	19.54	< LOQ	52.52	23.60	< LOQ	55.56	14.64	< LOQ	50.71
(C6H6)H+	79.051	1.21	0.69	1.72	1.04	0.52	1.65	0.91	0.52	1.38	1.45	0.49	1.97
(C6H8)H+	81.070	23.64	15.81	33.83	22.56	15.77	28.45	22.56	16.04	28.45	21.05	15.76	34.42
(C6H10)H+	83.079	7.99	< LOQ	12.87	7.33	< LOQ	12.03	< LOQ	< LOQ	10.87	9.02	< LOQ	13.29
(C5H8O)H+	85.059	6.78	4.53	8.51	6.23	4.19	7.81	5.99	4.26	7.70	6.38	4.19	7.82
(C6H12)H+	85.103	13.91	9.90	19.67	12.94	6.28	19.08	10.83	6.42	17.57	14.41	6.76	19.99
(C5H10O)H+	87.047	31.70	17.92	49.43	32.65	18.49	48.42	33.71	20.78	48.42	26.70	15.16	48.60
(C4H8S)H+	89.046	0.45	0.36	0.64	0.44	0.34	0.65	0.43	0.35	0.57	0.53	0.32	0.73
(C4H10S)H+	91.056	0.94	0.76	1.14	0.95	0.75	1.13	0.95	0.74	1.03	1.08	0.80	1.23
(C7H8)H+	93.054	< LOQ	< LOQ	< LOQ	< LOQ	< LOQ	< LOQ	< LOQ	< LOQ	< LOQ	< LOQ	< LOQ	< LOQ
(C6H6O)H+	95.055	< LOQ	< LOQ	< LOQ	< LOQ	< LOQ	< LOQ	< LOQ	< LOQ	< LOQ	< LOQ	< LOQ	< LOQ
(C6H8O)H+	97.089	10.26	7.62	13.59	9.58	7.41	13.04	10.15	7.83	11.83	9.29	7.10	13.44
(C6H12O)H+	101.059	< LOQ	< LOQ	< LOQ	< LOQ	< LOQ	< LOQ	< LOQ	< LOQ	< LOQ	< LOQ	< LOQ	< LOQ
(C8H8)H+	105.063	22.13	18.10	30.36	21.82	15.91	29.55	21.39	16.21	25.27	22.61	15.14	32.15
(C8H10)H+	107.064	0.57	< LOQ	1.63	0.45	< LOQ	1.75	0.27	< LOQ	1.36	1.03	< LOQ	2.67
(C8H12)H+	109.092	1.40	< LOQ	1.78	1.36	< LOQ	1.68	1.38	< LOQ	1.68	1.24	< LOQ	1.66
(C7H10O)H+	111.100	< LOQ	< LOQ	< LOQ	< LOQ	< LOQ	< LOQ	< LOQ	< LOQ	< LOQ	< LOQ	< LOQ	< LOQ
(C9H10)	119.077	< LOQ	< LOQ	< LOQ	< LOQ	< LOQ	< LOQ	< LOQ	< LOQ	< LOQ	< LOQ	< LOQ	< LOQ
(C8H8O)H+	121.062	20.11	15.77	26.13	20.40	16.38	23.72	19.29	15.19	23.59	21.72	17.76	23.99
(C7H6O2)H+	123.044	10.86	9.04	12.87	10.56	8.48	12.56	11.08	8.57	12.75	10.03	8.54	12.56
(C9H10O)H+	135.070	23.02	17.93	31.54	21.85	16.83	30.34	19.48	13.47	27.96	23.29	18.22	32.59
(C10H16)H+	137.133	2.75	0.13	5.55	2.55	0.92	5.17	2.42	1.02	5.44	2.73	0.78	4.58
(C10H12O)H+	149.056	< LOQ	< LOQ	< LOQ	< LOQ	< LOQ	< LOQ	< LOQ	< LOQ	< LOQ	< LOQ	< LOQ	< LOQ
(C10H22O)H+	159.094	< LOQ	< LOQ	11.70	< LOQ	< LOQ	16.17	< LOQ	< LOQ	13.45	< LOQ	< LOQ	17.60

ROOM AIR		Mono-infections											
Sum formula	Mass	All *S. pneumoniae* (n=40)			Asymptomatic *S. pneumoniae* (n=25)			Symptomatic *S. pneumoniae* (n=15)			All Rhinovirus (n=34)		
		Median	25-percentile	75-percentile	Median	25-percentile	75-percentile	Median	25-percentile	75-percentile	Median	25-percentile	75-percentile
(CH2O)H+	31.018	190.16	101.00	256.44	237.17	159.01	299.21	144.78	89.35	181.39	162.42	73.55	257.76
CH4O)H+	33.031	< LOQ	< LOQ	< LOQ	< LOQ	< LOQ	< LOQ	< LOQ	< LOQ	< LOQ	< LOQ	< LOQ	< LOQ
(H2S)H+	34.970	2.85	2.43	3.30	2.76	2.29	2.94	3.26	2.79	3.63	3.14	2.70	3.34
(C3H4)H+	41.034	16597.07	8862.89	26164.01	25257.64	15375.04	35441.85	10766.98	7894.24	16109.64	15814.17	6123.80	25172.24
(C3H6)H+	43.046	11602.23	6440.02	20270.76	20057.78	10920.55	26020.38	8294.74	6078.08	11515.46	11555.28	4593.96	18460.30
(CO2)H+	44.997	41.88	32.55	64.73	46.18	41.44	71.17	32.41	22.40	41.07	31.51	27.12	53.99
(C2H4O)H+	45.027	49.81	32.19	114.19	92.00	47.08	120.16	44.59	28.32	50.89	52.94	24.53	81.62
(CH2O2)H+	47.017	45.36	41.35	51.09	47.18	44.57	54.34	41.63	38.88	46.30	44.47	40.75	48.70
(C2H6O)H+	47.056	< LOQ	< LOQ	< LOQ	< LOQ	< LOQ	< LOQ	< LOQ	< LOQ	< LOQ	< LOQ	< LOQ	< LOQ
(CH4S)H+	49.011	6.55	5.54	8.89	7.70	5.90	10.50	6.12	5.07	6.73	6.94	5.50	7.88
(C3H4O)H+	57.041	< LOQ	< LOQ	< LOQ	< LOQ	< LOQ	0.21	< LOQ	< LOQ	< LOQ	< LOQ	< LOQ	< LOQ
(C4H8)H+	57.072	99.21	59.32	153.84	112.41	59.62	160.13	80.13	61.68	113.36	132.80	73.01	169.98
(C3H6O)H+	59.049	7.81	4.19	17.01	16.81	7.41	24.58	5.38	3.63	7.71	8.31	3.87	14.89
(C2H4O2)H+	61.031	15.07	10.43	18.63	15.41	9.52	17.28	14.73	11.60	18.94	16.53	13.54	20.63
(C3H8O)H+	61.066	602.01	379.24	1422.66	1353.90	524.94	1805.32	419.15	306.94	568.98	814.83	332.35	1204.24
(C2H6S)H+	63.025	< LOQ	< LOQ	19.99	< LOQ	< LOQ	30.65	< LOQ	< LOQ	< LOQ	< LOQ	< LOQ	21.61
(C5H6)H+	67.057	12.30	9.10	15.58	13.91	10.39	16.07	9.50	8.64	13.60	13.28	9.91	15.66
(C5H8)H+	69.070	< LOQ	< LOQ	1.69	1.27	< LOQ	2.15	< LOQ	< LOQ	0.51	0.10	< LOQ	2.91
(C4H6O)H+	71.043	0.35	0.26	0.48	0.39	0.24	0.53	0.33	0.28	0.37	0.38	0.26	0.47
(C5H10)H+	71.082	15.16	11.16	25.02	15.60	10.40	27.66	13.44	12.13	19.72	22.97	14.32	28.89
(C4H8O)H+	73.067	13.97	< LOQ	55.80	36.80	< LOQ	84.88	4.71	< LOQ	23.89	41.70	< LOQ	66.07
(C6H6)H+	79.051	1.08	0.53	1.63	1.30	0.68	1.65	0.83	0.41	1.52	1.33	0.65	1.89
(C6H8)H+	81.070	22.56	16.37	31.17	24.40	22.25	36.99	16.39	13.89	21.70	23.72	16.83	33.85
(C6H10)H+	83.079	7.72	< LOQ	10.78	9.23	< LOQ	13.43	< LOQ	< LOQ	7.77	7.41	< LOQ	13.63
(C5H8O)H+	85.059	5.92	4.33	9.06	7.46	4.38	9.06	5.04	3.99	6.80	7.22	4.90	8.31
(C6H12)H+	85.103	11.47	6.99	20.91	13.81	7.16	21.25	9.90	6.69	16.05	15.23	9.82	22.86
(C5H10O)H+	87.047	29.99	19.84	62.70	44.58	22.42	66.86	23.85	7.01	31.97	38.93	17.60	58.40
(C4H8S)H+	89.046	0.44	0.35	0.64	0.44	0.39	0.65	0.44	0.33	0.62	0.56	0.44	0.88
(C4H10S)H+	91.056	0.93	0.76	1.12	0.96	0.78	1.16	0.85	0.76	0.95	1.09	0.87	1.26
(C7H8)H+	93.054	< LOQ	< LOQ	< LOQ	< LOQ	< LOQ	< LOQ	< LOQ	< LOQ	< LOQ	< LOQ	< LOQ	< LOQ
(C6H6O)H+	95.055	< LOQ	< LOQ	3.64	< LOQ	< LOQ	3.71	< LOQ	< LOQ	< LOQ	< LOQ	< LOQ	< LOQ
(C6H8O)H+	97.089	9.51	7.53	14.90	10.87	9.38	15.39	8.23	6.50	9.51	10.76	7.73	14.37
(C6H12O)H+	101.059	< LOQ	< LOQ	< LOQ	< LOQ	< LOQ	< LOQ	< LOQ	< LOQ	< LOQ	< LOQ	< LOQ	< LOQ
(C8H8)H+	105.063	21.94	16.37	29.07	25.06	21.05	30.46	16.30	12.83	23.18	22.03	15.95	29.02
(C8H10)H+	107.064	0.33	< LOQ	1.64	0.38	< LOQ	1.63	0.28	< LOQ	1.56	1.45	0.48	2.22
(C8H12)H+	109.092	1.42	< LOQ	1.62	1.57	1.40	2.04	< LOQ	< LOQ	1.27	1.43	< LOQ	1.91
(C7H10O)H+	111.100	< LOQ	< LOQ	< LOQ	< LOQ	< LOQ	< LOQ	< LOQ	< LOQ	< LOQ	< LOQ	< LOQ	< LOQ
(C9H10)	119.077	< LOQ	< LOQ	< LOQ	< LOQ	< LOQ	23.89	< LOQ	< LOQ	< LOQ	< LOQ	< LOQ	< LOQ
(C8H8O)H+	121.062	22.79	16.00	32.96	23.76	16.14	35.43	20.66	15.93	31.98	20.78	16.06	24.74
(C7H6O2)H+	123.044	11.16	9.20	12.20	12.14	10.69	14.43	9.70	7.78	11.06	11.04	8.64	13.22
(C9H10O)H+	135.070	22.51	15.07	33.15	23.28	15.18	33.15	21.79	14.98	24.86	24.65	12.85	29.82
(C10H16)H+	137.133	3.26	0.57	6.35	4.86	1.78	6.42	1.64	< LOQ	3.35	4.13	0.67	6.54
(C10H12O)H+	149.056	< LOQ	< LOQ	< LOQ	< LOQ	< LOQ	< LOQ	< LOQ	< LOQ	< LOQ	< LOQ	< LOQ	< LOQ
(C10H22O)H+	159.094	< LOQ	< LOQ	18.12	< LOQ	< LOQ	17.48	< LOQ	< LOQ	18.75	11.44	< LOQ	19.33

ROOM AIR		Coinfections								
Sum formula	Mass	*H. influenzae* + *S. pneumoniae* (n=24)			*H. influenzae* + Rhinovirus (n=16)			*S. pneumoniae* + Rhinovirus (n=12)		
		Median	25-percentile	75-percentile	Median	25-percentile	75-percentile	Median	25-percentile	75-percentile
(CH2O)H+	31.018	210.74	147.20	293.27	181.39	82.57	263.68	280.77	80.63	320.24
CH4O)H+	33.031	< LOQ	< LOQ	< LOQ	< LOQ	< LOQ	< LOQ	< LOQ	< LOQ	< LOQ
(H2S)H+	34.970	3.16	2.53	3.43	2.91	2.54	3.24	3.20	2.48	3.62
(C3H4)H+	41.034	17376.61	11040.11	27674.16	14101.30	6806.28	26265.27	25038.32	6963.21	28762.61
(C3H6)H+	43.046	12529.16	8320.00	20048.41	10339.24	5042.01	19244.14	18587.67	4840.82	20333.41
(CO2)H+	44.997	35.52	26.41	66.68	46.99	25.34	60.70	45.78	23.77	62.39
(C2H4O)H+	45.027	61.52	36.35	91.09	49.14	28.25	76.40	73.40	28.70	92.60
(CH2O2)H+	47.017	43.71	41.03	48.83	45.49	41.31	50.31	44.90	41.61	51.51
(C2H6O)H+	47.056	< LOQ	< LOQ	< LOQ	< LOQ	< LOQ	< LOQ	< LOQ	< LOQ	< LOQ
(CH4S)H+	49.011	7.37	6.00	7.66	6.92	6.12	8.38	7.52	5.48	9.32
(C3H4O)H+	57.041	< LOQ	< LOQ	< LOQ	< LOQ	< LOQ	0.14	< LOQ	< LOQ	< LOQ
(C4H8)H+	57.072	113.26	74.52	151.92	104.05	58.04	170.20	85.72	43.83	119.18
(C3H6O)H+	59.049	9.70	5.23	17.01	8.35	3.81	15.53	13.86	2.49	19.74
(C2H4O2)H+	61.031	17.07	11.08	19.69	12.52	11.34	16.28	10.85	10.05	14.57
(C3H8O)H+	61.066	876.36	523.03	1408.91	551.68	362.74	1025.00	998.39	182.69	1434.40
(C2H6S)H+	63.025	< LOQ	< LOQ	3.19	< LOQ	< LOQ	19.38	< LOQ	< LOQ	2.75
(C5H6)H+	67.057	13.73	9.56	16.19	9.99	8.54	14.83	9.51	7.79	13.75
(C5H8)H+	69.070	0.81	< LOQ	2.69	< LOQ	< LOQ	1.39	< LOQ	< LOQ	0.26
(C4H6O)H+	71.043	0.36	0.26	0.47	0.32	0.25	0.47	0.28	0.22	0.41
(C5H10)H+	71.082	20.52	15.90	26.12	15.32	9.35	26.90	14.25	8.15	20.36
(C4H8O)H+	73.067	46.12	3.54	60.52	15.92	< LOQ	47.76	27.66	< LOQ	46.70
(C6H6)H+	79.051	1.32	0.82	1.87	1.03	0.54	1.67	0.71	0.43	1.27
(C6H8)H+	81.070	21.48	14.52	27.95	25.26	16.91	32.69	20.21	14.85	25.74
(C6H10)H+	83.079	9.70	< LOQ	12.49	3.61	< LOQ	11.70	4.13	< LOQ	9.44
(C5H8O)H+	85.059	7.47	4.69	8.73	6.03	4.01	8.62	4.87	3.28	7.82
(C6H12)H+	85.103	14.74	8.54	19.31	12.58	7.15	19.41	9.07	5.17	12.78
(C5H10O)H+	87.047	24.44	14.70	57.03	30.94	19.17	46.57	28.22	17.97	42.93
(C4H8S)H+	89.046	0.53	0.41	0.70	0.46	0.35	0.65	0.40	0.32	0.49
(C4H10S)H+	91.056	1.03	0.69	1.11	0.88	0.72	1.13	0.74	0.66	0.98
(C7H8)H+	93.054	< LOQ	< LOQ	< LOQ	< LOQ	< LOQ	< LOQ	< LOQ	< LOQ	< LOQ
(C6H6O)H+	95.055	< LOQ	< LOQ	< LOQ	< LOQ	< LOQ	< LOQ	< LOQ	< LOQ	< LOQ
(C6H8O)H+	97.089	11.11	7.70	14.00	10.82	7.59	14.20	8.97	6.38	12.12
(C6H12O)H+	101.059	< LOQ	< LOQ	< LOQ	< LOQ	< LOQ	< LOQ	< LOQ	< LOQ	< LOQ
(C8H8)H+	105.063	24.18	16.38	29.67	20.32	12.28	24.24	19.87	13.52	22.78
(C8H10)H+	107.064	1.08	0.27	2.20	0.44	< LOQ	2.15	0.16	< LOQ	0.69
(C8H12)H+	109.092	1.41	< LOQ	1.65	0.74	< LOQ	1.75	< LOQ	< LOQ	1.44
(C7H10O)H+	111.100	< LOQ	< LOQ	< LOQ	< LOQ	< LOQ	< LOQ	< LOQ	< LOQ	< LOQ
(C9H10)	119.077	< LOQ	< LOQ	< LOQ	< LOQ	< LOQ	< LOQ	< LOQ	< LOQ	< LOQ
(C8H8O)H+	121.062	19.89	16.32	22.67	18.40	15.97	20.97	17.18	15.33	21.45
(C7H6O2)H+	123.044	10.81	9.12	12.83	10.32	8.53	12.60	9.86	8.58	11.52
(C9H10O)H+	135.070	25.74	19.99	33.30	24.02	14.60	31.25	20.99	10.79	32.03
(C10H16)H+	137.133	1.47	< LOQ	4.16	4.26	1.72	5.49	1.09	0.50	4.55
(C10H12O)H+	149.056	< LOQ	< LOQ	< LOQ	< LOQ	< LOQ	< LOQ	< LOQ	< LOQ	2.98
(C10H22O)H+	159.094	11.57	< LOQ	17.88	< LOQ	< LOQ	14.42	< LOQ	< LOQ	12.12

	Acetone	Acetic acid	Dimethyl sulphide	Pentanal	Limonene
**LOD [ppbV]**	0.02	2.36	3.51	9.54	1.99
**LOQ [ppbV]**	0.71	3.05	12.95	22.79	8.34

Table S4: LOD and LOQ of the five VOCs of interest.
